# Vaginal Microbiota Composition and Its Relationship with Fertility in Repeat Breeder Dairy Cows

**DOI:** 10.3390/biology15090668

**Published:** 2026-04-23

**Authors:** Erika J. Félix-Santiago, Delia X. Vega-Manríquez, Jorge Flores-Sánchez, Carlos A. Eslava-Campos, Ulises Hernández-Chiñas, Andrea García-Mendoza, Milagros González-Hernández, César A. Rosales-Nieto

**Affiliations:** 1 Facultad de Agronomía y Veterinaria, Universidad Autónoma San Luis Potosí, San Luis Potosí 78321, Mexico; 2 Laboratorio de Especialidades Médicas, Facultad de Medicina, Universidad Autónoma San Luis Potosí, San Luis Potosí 78290, Mexico; 3 Unidad Periférica de Investigación Básica y Clínica en Enfermedades Infecciosas, División de Investigación/Departamento de Salud Pública, Laboratorio de Patogenicidad Bacteriana, Unidad de Hemato-Oncología e Investigación, Hospital Infantil de México Federico Gómez, Facultad de Medicina, Universidad Nacional Autónoma de México, Dr. Márquez 162, Col. De los Doctores, Ciudad de México 06720, Mexico

**Keywords:** microbiota, progesterone, glucose, fertility rate, repeat breeder cow syndrome

## Abstract

Milk production in dairy herds is determined by both intrinsic and extrinsic factors, with reproductive efficiency serving as a primary determinant. Infectious, nutritional, and management-related challenges can reduce this efficiency. Following parturition, cows are more susceptible to clinical disorders due to a temporary loss of integrity in the cervix, vagina, and vulva, which allows environmental bacteria to ascend and alter the vaginal microbiota. These microbial changes may disrupt endocrine responses related to conception and contribute to repeat breeder cow syndrome (RBCS), which is defined as failure to conceive after three or more inseminations. This study investigated associations among cultivable vaginal bacteria, circulating progesterone and glucose concentrations, and reproductive performance in 30 fourth-parity Holstein cows with a body condition score of 3.5. Cows were classified by reproductive history as repeat breeders (RBCS; n = 14) or controls (CTL; n = 16). Vaginal mucosal samples were collected at insemination and cultured on blood agar and MacConkey media under aerobic and microaerobic conditions. Bacterial identification was conducted using Gram staining and standard biochemical assays. Blood samples were collected at insemination, on day 5 post-insemination, and every two days thereafter to measure progesterone and glucose concentrations. Fertility outcomes were analyzed using PROC GLIMMIX, and hormonal data were analyzed using mixed models with repeated measures. The bacterial genera identified included *Bacillus*, *Escherichia coli*, *Staphylococcus*, *Klebsiella*, *Proteus*, *Streptococcus*, and *Actinomyces*. Progesterone and glucose concentrations did not differ significantly between groups (*p* > 0.05). However, the fertility rate (*p* < 0.05; CTL:87.50% vs. RBCS:57.14%) and number of attempts to conceive (*p* < 0.001; CTL:2.5 vs. RBCS:6.7) differed statistically between treatments. A higher prevalence of *S. hyicus* was detected in RBCS cows, and *E. coli*, *S. hyicus*, and *Proteus* spp. were more frequently detected in non-pregnant cows. These findings suggest that the identified cultivable vaginal bacteria are associated with reproductive status in dairy cows.

## Simple Summary

Milk production in dairy herds is partially determined to reproductive efficiency. Following parturition, cows are more susceptible to infections, as opportunistic bacteria can colonize the reproductive tract and alter the composition of the vaginal microbiota. Such alterations may disrupt endocrine function and contribute to the development of repeat breeder cow syndrome (RBCS), defined as the failure to conceive after three or more artificial inseminations. In this study, we compared cultivable vaginal bacterial populations, progesterone and glucose concentrations, and fertility outcomes in Holstein cows diagnosed with or without RBCS. Although progesterone and glucose concentrations did not differ between groups, cows with RBCS exhibited reduced fertility, as evidenced by lower conception rates and a greater number of inseminations required to achieve pregnancy. Moreover, specific bacterial taxa were more frequently isolated from non-pregnant cows, suggesting that alterations in cultivable vaginal microbiota may be associated with impaired fertility.

## 1. Introduction

Reproductive efficiency is essential for the sustainability and profitability of dairy production systems; however, suboptimal fertility remains a significant global challenge [1]. Even minor reductions in reproductive performance can result in prolonged calving intervals, increased insemination costs, higher culling rates, and greater overall production expenses [2]. Repeat breeder cow syndrome (RBCS), defined as the failure to conceive after three or more consecutive artificial inseminations despite normal estrous cycles and the absence of clinical abnormalities, is a major cause of reproductive failure [3,4]. This syndrome is prevalent, economically impactful, and frequently affects cows during their peak productive years [3,4]. Reproductive inefficiency has traditionally been attributed to endocrine dysfunction, metabolic imbalances, infectious diseases, and management-related factors. During the peripartum period, disruption of the anatomical and physical barriers of the vulva, vagina, and cervix facilitates bacterial colonization of the reproductive tract, increasing the risk of both clinical and subclinical uterine diseases such as metritis and endometritis [5,6]. These diseases impair fertility by causing inflammatory damage to the endometrium and disrupting normal uterine function [7]. The development and persistence of uterine disease are determined by a complex interaction among bacterial load, microbial composition, and host immune competence [8,9]. Notably, emerging evidence suggests that reproductive failure can occur in the absence of clinically detectable pathology, indicating the involvement of more subtle, localized factors within the reproductive tract.

The female reproductive tract contains a complex and dynamic microbial ecosystem that interacts with host immune function, endocrine signaling, and tissue homeostasis [10]. The bovine vaginal microbiota is highly diverse, consisting of a wide range of aerobic and anaerobic bacterial species [11]. Its composition is shaped by factors such as parity, stage of the estrous cycle, metabolic status, nutrition, and environmental conditions [12,13,14]. Changes in vaginal microbial communities have been associated with postpartum uterine inflammation and reduced fertility, indicating that microbial dysbiosis may contribute to reproductive dysfunction [10]. However, the specific role of the vaginal microbiota in cows with reproductive inefficiency but without overt uterine disease remains insufficiently characterized. Several bacterial genera, including *Escherichia coli*, *Staphylococcus*, *Bacillus*, *Proteus*, and *Klebsiella*, are frequently isolated from the bovine vagina in both healthy and animals with reproductive disorders [15,16,17]. This finding suggests that their impact on fertility likely depends on factors such as relative abundance, strain-level pathogenicity, and interactions with other microorganisms and host physiology. Opportunistic pathogens such as *E. coli* and *Staphylococcus hyicus* possess virulence traits that can induce subclinical inflammation, compromise epithelial integrity, and alter local immune responses [15,18,19]. These mechanisms may adversely affect sperm viability, fertilization, or early embryonic development, even in the absence of clinical symptoms.

Endocrine and metabolic factors are central to the regulation of reproductive tract function and microbial ecology. Progesterone (P4) regulates luteal activity, endometrial receptivity, and the establishment of early pregnancy, while estrogens influence vaginal epithelial turnover, luminal pH, and the composition of microbial communities throughout the estrous cycle [20,21]. Metabolic status, often indicated by circulating glucose concentrations, is also vital for ovarian function, steroidogenesis, and immune competence [22]. Negative energy balance and reduced glucose availability can impair follicular development, disrupt gonadotropin secretion, and weaken immune defences, thereby increasing the risk of reproductive failure. Hormonal and metabolic signals also shape the reproductive tract microbiota, supporting a bidirectional relationship between host physiology and microbial communities [23,24,25]. Although interest in bovine reproductive microbiome is growing, most research has focused on the uterus during the postpartum period, with relatively little attention to the vaginal microbiota during breeding and early pregnancy [4]. Furthermore, few studies have simultaneously assessed vaginal microbial composition alongside systemic hormonal and metabolic profiles in cows with different reproductive histories [26,27]. An integrative approach is necessary to determine whether microbial alterations are secondary to systemic dysfunction or represent localized factors that directly impair fertility, particularly in cows with RBCS.

Current knowledge regarding differences in vaginal microbiota between cows with RBCS and fertile dairy cows is limited, particularly regarding whether these differences are associated with impaired fertility, independent of systemic hormonal and metabolic status, during the early post-insemination period. Additionally, the specific bacterial taxa that may contribute to reduced conception rates and increased insemination attempts are not well defined. The objective of this study was to characterize cultivable vaginal bacterial populations in RBCS and fertile control dairy cows and to evaluate their associations with circulating progesterone and glucose concentrations during the early post-insemination period. Fertility outcomes were also assessed to determine whether specific bacterial taxa were linked to reduced conception rates and a greater number of inseminations required to achieve pregnancy. It was hypothesized that cows with RBCS would display distinct vaginal microbial profiles associated with impaired fertility, independent of systemic endocrine and metabolic status. Addressing this knowledge gap will enhance understanding of host–microbiota interactions in bovine reproduction and may help identify microbial targets for improving reproductive efficiency in dairy herds.

## 2. Materials and Methods

All experimental procedures were conducted in accordance with the Technical Specifications for the Production, Care, and Use of Laboratory Animals established by the Official Mexican Standards [28]. The study protocol was approved by the Institutional Animal Care and Use Committee of the University of San Luis Potosí (CICUAE/2023/01).

The study was carried out on a commercial dairy farm located in central Mexico (21°73′ N, 100°96′ W), characterized by a semi-arid temperate climate. The region has an average annual temperature of 16.7 °C and annual precipitation of approximately 361 mm. Relative humidity ranges from 45% in April to 90% during the rainy season. The herd consisted of approximately 3000 Holstein and Jersey cows, of which around 2000 were in lactation.

### 2.1. Experimental Design

Thirty cows were assigned to two experimental groups based on body condition score (BCS; scale 1–5; [29]) and reproductive history. This sample size is consistent with previous studies in bovine reproductive physiology and animal production, where group sizes of 20–40 animals are considered sufficient to detect biologically meaningful differences under controlled conditions [12,13,26,30,31]. Reproductive history parameters included days open, number of lactations, and number of inseminations. All cows had a BCS of 3.5 and were in their fourth lactation. Cows diagnosed with repeat breeder cow syndrome (RBCS; n = 14) had a mean of 201 days open (range: 154–269) and an average of 5 inseminations (range: 4–11). Control cows (CTL; n = 16) had a mean of 74 days open (range: 60–112) and no prior inseminations, except for one cow that had undergone a single insemination. On this farm, cows are typically first inseminated at approximately 70 days postpartum.

### 2.2. Housing Conditions, Feeding Regimen, and Management

Cows were housed in 30 × 30 m^2^ pens with approximately 85% dirt and 15% concrete flooring. Animals were grouped by lactation number; therefore, all experimental cows were maintained within a single pen. The diet was formulated to meet maintenance and production requirements, providing 2.2 Mcal of metabolizable energy and 14.1% crude protein. It consisted of hay, fresh alfalfa, rolled corn, cottonseed, corn silage, molasses, and a mineral premix. These ingredients were mixed using a feed mixer wagon and delivered along a concrete feed bunk within the pen. Cows had ad libitum access to both feed and water, and feed availability was maintained continuously. All animals were vaccinated biannually against bovine viral rhinotracheitis, bovine viral diarrhea (BVD; strains 1 and 2), bovine respiratory syncytial virus (BRSV), clostridial diseases, and pneumonic pasteurellosis. No anthelmintic treatments were administered during the study period.

### 2.3. Estrus Detection, Vaginal Sample, Insemination, and Fertility

**Figure 1:**
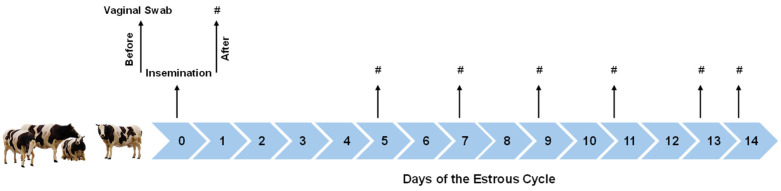
Schema of Experimental Design. Vaginal swabs and blood samples were collected according to the experimental design. Cows were classified as Repeat Breeder Cow Syndrome (RBCS) or not (Control: CTL) based on their reproductive history. Immediately prior to insemination (within one minute), a vaginal swab was collected from each cow. Blood samples (#) were obtained on the day of insemination (day 0), on day 5 post-insemination, and thereafter every 2 days for a total of 14 days to assess hormonal and metabolic profiles.

Estrus detection was performed following milking using pedometer-based activity monitoring, with oversight by trained farm personnel. Estrus status was initially identified using commercial herd management software (DAIRYCOMP^®^; Valley Agricultural Software, Visalia, CA, USA) and subsequently confirmed by a trained reproductive technician. Once estrus was verified, artificial insemination (AI) was performed. The AI procedure included semen preparation and thawing, sanitation of the vulvar area, insertion of the AI gun, and deposition of semen into the uterine body after passage through the cervix. Cows did not exhibit estrus simultaneously; therefore, inseminations were conducted on different days. Semen from multiple bulls was used, and if pregnancy was not achieved after two inseminations, the semen source was changed, with a general decline in genetic merit over successive services. Immediately prior to insemination, a vaginal mucosal sample was collected from each cow using a sterile, disposable culture swab (MINITUBE^®^, Tiefenbach, Germany; Figure 1). Pregnancy diagnosis was performed 35 days post-insemination by ultrasonography and confirmed by transrectal palpation conducted by the reproductive technician.

### 2.4. Cultivable Vaginal Bacteria

Samples were collected approximately one minute prior to AI [13]. Following aseptic preparation of the perineal region using disinfectant and disposable paper towels, a sterile, disposable culture swab (MINITUBE^®^, Tiefenbach, Germany) was introduced into the vaginal canal. After gentle separation of the vulvar labia and careful avoidance of contact with the external genitalia to minimize contamination, a sample of the vaginal mucosa was obtained using rotational movements. The swab was then withdrawn with equal care and immediately placed into AMIES agar transport medium and stored at 4 °C until processing [13]. In the microbiology laboratory, samples were refrigerated and cultured within 24 h of collection. Swabs were streaked onto blood agar and MacConkey agar plates and incubated at 37 °C for 24 and 48 h under both aerobic and microaerobic conditions [32]. Isolated colonies were identified by Gram staining and standard biochemical assays to determine the bacterial genus and species [32]. Gram-positive bacteria were characterized using catalase and coagulase tests, growth on mannitol salt agar, the Voges–Proskauer reaction, and carbohydrate fermentation profiles. Gram-negative bacteria were identified using IMViC-related and complementary biochemical tests, including sulfide–indole–motility (SIM), Simmons citrate utilization, urease activity, methyl red–Voges–Proskauer (MR–VP), and Kligler iron agar (KIA) reactions [32].

### 2.5. Blood Samples

Blood samples were collected on the day of insemination (day 0), on day 5 post-insemination, and every two days thereafter for 14 days via coccygeal venipuncture. Samples were drawn into 6 mL additive-free Vacutainer^®^ tubes (Vacutainer^®^, Becton Dickinson, NE, USA). After collection, blood samples were centrifuged at 3500 rpm for 10 min within 30 min of sampling. Plasma was separated, aliquoted into three portions in Eppendorf^®^ tubes, and stored at −80 °C until analysis. Plasma glucose concentrations were measured using a turbidimetric method with an A25 BioSystems^®^ analyzer and reagent kit (catalog number 12503; BioSystems S.A., Barcelona, Spain), with a detection limit of 1.6 mg/dL (0.08 mmol/L). Plasma progesterone concentrations were quantified using a chemiluminescent immunoassay on an IMMULITE 1000 system (catalog number LKPW1; Siemens Healthcare, New York, NY, USA), according to the manufacturer’s instructions. The assay calibration range was 0.20–20 ng/mL (0.64–64 nmol/L), with a sensitivity of 0.20 ng/mL (0.64 nmol/L). Analytical accuracy was ensured by running commercially available quality control samples at three concentration levels (0.64, 7.5, and 20.9 ng/mL) daily prior to sample analysis. Assays were accepted when the coefficient of variation (CV) was less than 10% at each control level and when replicates were within the manufacturer’s specified ranges. Prior to analysis, plasma samples were thawed at room temperature and analyzed in duplicate. Concentrations were calculated as the mean of duplicate measurements. The intra-assay coefficient of variation was determined, and all reported values had CVs below 10%.

### 2.6. Statistical Analysis

All analyses were performed in SAS 9.4 [33]. Each animal was considered an experimental unit. Data distribution normality was assessed with the Shapiro–Wilk test (PROC UNIVARIATE). P4 and glucose concentrations were analyzed using mixed models (PROC MIXED) with treatment and sampling date as fixed effects and cow ID as a random effect. Fertility (pregnant vs. no-pregnant) and cultivable vaginal bacteria identified were analyzed using the generalized linear mixed model procedures with a binomial distribution and logit link function (PROC-GLIMMIX). Treatment was the fixed effect in the model. For fertility, the bacteria identified were included as covariates in the analysis. Significant differences among means were determined using LS-MEANS, with *p* < 0.05 considered statistically significant.

## 3. Results

### 3.1. Vaginal Bacteria

A total of 70 bacterial strains were initially isolated from cows in the study. After re-isolation procedures, 49 strains were successfully recovered, consisting of 22 Gram-negative and 27 Gram-positive isolates. Among these, 23 strains were derived from RBCS cows and 26 from CTL cows. Eight bacterial genera were identified in both experimental groups, including *Escherichia coli*, *Bacillus* spp., and coagulase-positive *Staphylococcus* (Table 1). CTL cows demonstrated a higher prevalence of *E. coli* and *Proteus* spp. compared to RBCS cows (*p* < 0.05). Conversely, RBCS cows exhibited a significantly higher prevalence of *Staphylococcus hyicus* than CTL cows (*p* < 0.01). The genera *Klebsiella* and *Shigella* were detected exclusively in RBCS cows, but these differences were not statistically significant (*p* > 0.05). *Bacillus spp*. were more frequently observed in CTL cows than in RBCS cows, although this difference was also not statistically significant (*p* > 0.05). Detailed prevalence data are provided in Table 1.

### 3.2. Progesterone (P4)

**Figure 2:**
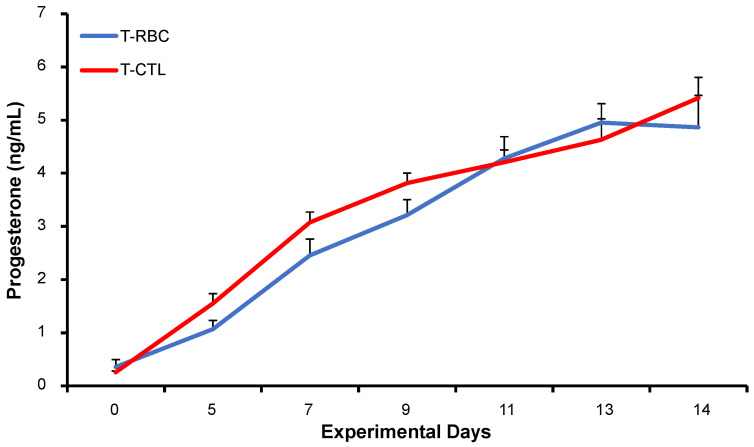
Mean (±SEM) circulating progesterone concentration in Holstein cows in their 4th parity with Repeat Breeder Cow Syndrome (RBCS; blue solid line) or those without the syndrome (CTL; red solid line). Day 0 represents the day when the cow was inseminated. Experimental days represent the dates on which the blood samples were collected.

P4 secretion profiles exhibited a comparable temporal pattern in both treatment groups (*p* > 0.05; Figure 2). On the day of insemination (day 0), mean P4 concentrations measured 0.35 ng/mL in RBCS cows and 0.25 ng/mL in CTL cows. Subsequently, P4 concentrations increased throughout the post-insemination period, reaching 5.73 ng/mL in RBCS cows and 4.98 ng/mL in CTL cows by day 14. P4 concentrations varied significantly across sampling days (*p* < 0.001; Figure 2), consistent with the physiological progression of luteal development following insemination. No significant differences were detected between treatment groups.

### 3.3. Glucose

**Figure 3:**
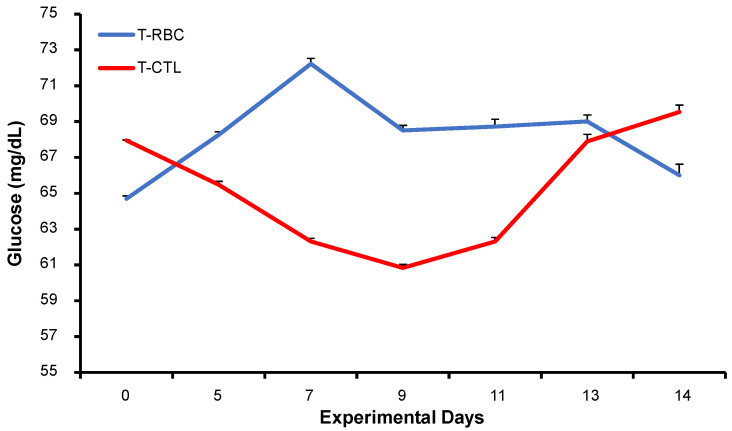
Mean (±SEM) circulating glucose concentration in Holstein cows in their 4th parity with Repeat Breeder Cow Syndrome (RBCS; blue solid line) or those without the syndrome (CTL; red solid line). Day 0 represents the day when the cow was inseminated. Experimental days represent the dates on which the blood samples were collected.

Circulating glucose levels did not differ between treatments across the experimental period (*p* > 0.05; Figure 3). Glucose secretion levels did not differ among sampling days (*p* > 0.05; Figure 3). The mean concentration during the 14 days post-insemination was 66.11 mg/dL for the RBCS and 66.91 mg/dL for CTL.

### 3.4. Fertility

The fertility rate differed statistically between treatments (*p* < 0.05; Table 2), with 14 of 16 cows in the CTL group becoming pregnant (87.50%) compared with 8 of 14 in the RBCS group (57.14%). The number of attempts to conceive also differed between treatments, averaging 6.7 (range 5–13) for RBCS cows versus 2.5 (range 1–4) for CTL cows (*p* < 0.001; Table 2). Regarding the bacteria present in cows that became pregnant, there was a difference in the presence of *S. hyicus* and *E. coli* in non-pregnant cows (*p* < 0.001; Table 3).

## 4. Discussion

This study presents novel evidence that links cultivable vaginal bacteria to fertility outcomes in dairy cows, independent of systemic progesterone (P4) and glucose dynamics during the early post-insemination period. While P4 profiles and glucose concentrations were similar between treatment groups, significant differences in reproductive performance were observed. These findings indicate that local conditions within the reproductive tract, particularly microbial ecology, are critical determinants of fertility. The study’s limitations include a relatively small sample size, the absence of semen-associated microbiota characterization, and reliance on culture-based microbiological methods, which may underestimate overall microbial diversity. Nonetheless, these limitations do not diminish the scientific significance of the results. Instead, this research offers a focused and practical contribution to understanding the microbial component of RBCS and provides a foundation for future investigations utilizing more comprehensive, culture-independent microbiome methodologies.

### 4.1. Cultivable Vaginal Bacteria and Its Relationship with Fertility

The cultivable vaginal microbiota in both treatment groups was dominated by *Escherichia coli*, *Bacillus spp*., and coagulase-positive *Staphylococcus*, consistent with previous reports describing a diverse, non-*Lactobacillus*-dominated bovine vaginal ecosystem [17]. Despite this shared core microbiota, significant treatment-related differences in the prevalence of specific bacterial genera were observed, which are likely biologically relevant to fertility outcomes. Across all cows, regardless of pregnancy status, CTL cows exhibited a higher prevalence of *E. coli* and *Proteus* spp., while RBCS cows demonstrated a significantly higher prevalence of *Staphylococcus hyicus*. Analysis by pregnancy status revealed that *S. hyicus* and *E. coli* were significantly associated with non-pregnant cows. These findings suggest that specific bacterial taxa, rather than overall bacterial presence, may have a disproportionate impact on reproductive success.

The increased prevalence of *E. coli* in CTL cows aligns with findings by Moore et al. [31], who reported higher abundance of this species in healthy Holstein and Jersey cows compared to animals with purulent vaginal discharge associated with postpartum inflammatory conditions. Conversely, *S. hyicus* was more frequently isolated from RBCS cows in the present study, which contrasts with observations in Criollo Limonero cows, where this species was more prevalent in healthy individuals [34]. At the genus level, *Staphylococcus* has been reported to be more abundant in healthy cows than in those with clinical or subclinical metritis [35,36], and is commonly identified as part of the normal vaginal microbiota in both healthy cows and those with purulent vaginal discharge [37]. These apparently contradictory findings indicate that *S. hyicus* may act as a commensal in some contexts and as an opportunistic pathogen in others [38]. *S. hyicus* is known to produce exfoliative toxins and induce epithelial inflammation [39]. Its increased prevalence in RBCS cows, particularly among non-pregnant animals, supports the hypothesis that subclinical vaginal inflammation may impair sperm transport, disrupt uterine immune tolerance, or compromise early embryonic survival. While emerging evidence suggests that semen-associated microbiota may also influence reproductive outcomes, this aspect was beyond the scope of the present study and requires targeted investigation to clarify its role in shaping vaginal microbial dynamics and fertility [40]. Furthermore, inter-individual variability in microbial composition, influenced by genetic background, nutrition, management practices, and environmental conditions, may contribute to the divergent roles observed for specific taxa [41,42].

Previous studies have linked *Proteus* spp. with abnormal vaginal mucus, often alongside *Fusobacterium necrophorum* and *Trueperella pyogenes* [43], although *Proteus* spp. has not been consistently classified as a primary uterine pathogen [44]. The higher prevalence of *Proteus* spp. in CTL cows observed in this study suggests that, in the absence of synergistic pathogenic interactions, this genus may not negatively impact fertility. This finding highlights the importance of microbial interactions, rather than the presence of individual taxa alone, in determining reproductive outcomes. Collectively, these results emphasize the context-dependent nature of the bovine vaginal microbiota and its modulation by host and environmental factors. Given the limitations of culture-based approaches, future studies utilizing high-resolution, culture-independent techniques such as 16S rRNA gene sequencing and metagenomics are necessary to more comprehensively characterize microbial community structure and function [35].

### 4.2. Progesterone Profiles and Glucose Levels Do Not Explain Fertility Differences

Circulating progesterone (P4) concentrations followed the expected post-insemination profile in both groups, increasing progressively and reaching luteal-phase levels by day 14, with no significant differences between treatments. This finding does not support the hypothesis that cows with RBCS exhibit impaired luteal function and is consistent with previous reports demonstrating similar P4 concentrations in pregnant and non-pregnant cows at the time of insemination [45]. Given the well-established role of inadequate P4 during early diestrus in embryonic loss [46], the similarity observed in the present study suggests that luteal insufficiency was unlikely to account for the differences in fertility outcomes. Nevertheless, P4 may still indirectly influence reproductive success through interactions with the vaginal microbiota, as microbial composition varies across the estrous cycle, likely under hormonal regulation [13,47]. Additional endocrine mediators, including prostaglandin F2α and estradiol, may further modulate microbial dynamics by influencing immune responses and vaginal pH [48,49]. Incorporating these hormonal parameters in future studies would provide a more integrated understanding of endocrine–microbiota interactions in the reproductive tract.

Circulating glucose concentrations were also comparable between treatments throughout the experimental period. Although systemic energy status is more comprehensively reflected by multiple metabolic indicators, including glucose, insulin, non-esterified fatty acids, and β-hydroxybutyrate, the results of this study suggest similar metabolic conditions across groups. A slight, non-significant decrease in glucose was observed in RBCS cows on day 9 post-insemination; however, the absence of consistent differences indicates that metabolic insufficiency was not a primary determinant of reduced fertility. This interpretation is consistent with previous studies highlighting the importance of glucose availability for follicular development, estradiol synthesis, and ovulatory function [50,51]. It should be noted that blood samples were not collected under fasting conditions, reflecting standard management practices in high-producing dairy systems, which may limit the precision of metabolic assessment.

### 4.3. Fertility Outcomes and Reproductive Efficiency

Despite similar P4 profiles and circulating glucose concentrations, fertility outcomes differed markedly between treatments. CTL cows exhibited a significantly higher pregnancy rate and required fewer insemination attempts than cows with RBCS, underscoring the biological relevance of these differences. The prolonged time to conception observed in RBCS cows is consistent with impaired uterine receptivity and/or increased early embryonic loss, conditions that may be exacerbated by an unfavorable vaginal microbiota [10,52]. When reproductive outcomes were stratified by pregnancy status, a higher prevalence of *S. hyicus* and *E. coli* was observed in non-pregnant cows. This finding is consistent with previous studies linking *E. coli* to postpartum uterine disorders, such as metritis and endometritis, which are known to negatively affect fertility [6,53,54]. Certain *E. coli* strains possess virulence factors that enable endotoxin production, inducing inflammatory responses in endometrial cells and predisposing the uterus to secondary infections [48]. In addition, the anatomical proximity of the anus and vulva facilitates fecal contamination, which likely explains the frequent presence of *E. coli* within the bovine reproductive tract [55]. Collectively, these results support the concept that reproductive inefficiency may arise from subtle, subclinical alterations in the vaginal microbial ecosystem, even in the absence of overt clinical disease.

## 5. Conclusions and Perspectives

In conclusion, the differences in fertility observed between RBCS and CTL cows were not associated with systemic progesterone profiles or circulating glucose concentrations, but rather coincided with variations in cultivable vaginal microbiota. Specifically, a higher prevalence of *S. hyicus* was detected in RBCS cows, while *E. coli*, *S. hyicus*, and *Proteus* spp. were more frequently identified in non-pregnant animals. These findings suggest a potential association between specific bacterial taxa and reproductive outcomes; however, they do not establish causality. Given the substantial economic impact of reduced fertility in dairy systems, further research is warranted to characterize the pathogenic potential and strain-level diversity of *E. coli* and *S. hyicus* identified in this study. In addition, the application of molecular and high-throughput sequencing approaches would enable a more comprehensive characterization of the bovine vaginal microbiome across different parities and reproductive states.

## Figures and Tables

**Table 1:** Bacterial genus prevalence in Holstein cows in their 4th parity with Repeat Breeder Cow Syndrome (RBCS) or not (CTL). The data combined pregnant and non-pregnant cows. The data present the number of cows and the percentage with bacterial genus prevalence.

Bacteria	RBCS (%)	CTL (%)	*p*-Value
*Bacillus*	4 (28.6)	6 (37.8)	0.159
*Escherichia coli*	6 (42.8)	9 (56.7)	0.045
*Klebsiella*	1 (7.1)	0 (0.0)	0.970
*Proteus*	1 (7.1)	3 (18.9)	0.017
*Shigella*	0 (0.0)	1 (7.0)	0.970
*Staphylococcus coag*	4 (28.6)	5 (31.5)	0.642
*Staphylococcus hyicus*	4 (28.6)	2 (12.6)	0.005
*Streptococcus*	1 (7.1)	2 (12.6)	0.196

**Table 2:** Mean attempts of Inseminations and Fertility rate in Holstein cows in their 4th parity with Repeat Breeder Cow Syndrome (RBCS) or not (CTL).

	Treatment		
	RBCS	CTL	*p*-Value
n	14	16	
Mean attempts of Inseminations	6.7	2.5	<0.001
Fertility (%)	57	87	<0.05

**Table 3:** Bacterial genus prevalence in Holstein cows in their 4th parity, pregnant and non-pregnant. The data combined cows with Repeat Breeder Cow Syndrome (RBCS) and those without (CTL). The data present the number of cows and the percentage with bacterial genus prevalence.

Bacteria	Pregnant (%)	Non-Pregnant (%)	*p*-Value
*Bacillus*	8 (36.6)	2 (25.0)	0.12
*Escherichia coli*	9 (41.2)	6 (75.0)	0.001
*Klebsiella*	0 (0.0)	1 (12.5)	0.96
*Proteus*	4 (18.3)	0 (0.0)	0.96
*Shigella*	0 (0.0)	1 (12.5)	0.96
*Staphylococcus coag*	7 (32.0)	2 (25.0)	0.32
*Staphylococcus hyicus*	2 (9.1)	4 (50.0)	0.001
*Streptococcus*	2 (9.1)	1 (12.5)	0.49

## Data Availability

The data presented in the manuscript were part of Erika J. Félix-Santiago’s Thesis Project. The corresponding author can provide information upon request.
